# Impact of intraoperative packed red blood cell transfusion volume on prognosis in CRS: a propensity-matched study

**DOI:** 10.3389/fsurg.2025.1688163

**Published:** 2026-01-12

**Authors:** Jie Li, Youzhong Xing, Wenwen Chen, Ya Sun

**Affiliations:** 1 Department of Blood Transfusion, Central Hospital Affiliated to Shandong First Medical University, Jinan, China; 2 Medical Imaging Center, Central Hospital Affiliated to Shandong First Medical University, Jinan, China; 3 Department of Gastrointestinal Surgery, Central Hospital Affiliated to Shandong First Medical University, Jinan, China

**Keywords:** complications, cytoreductive surgery, hyperthermic intraperitoneal chemotherapy, intraoperative packed RBC transfusion, survival analysis

## Abstract

**Background:**

Cytoreductive surgery (CRS) with hyperthermic intraperitoneal chemotherapy (HIPEC) is an effective treatment for peritoneal metastasis but is associated with significant blood loss requiring intraoperative red blood cell (RBC) transfusion. Evidence suggests transfusions may impair immunity and worsen oncologic outcomes, yet the prognostic impact of intraoperative packed RBC transfusion (iPRBT), especially transfusion volume, in CRS/HIPEC remains unclear.

**Methods:**

We conducted a single-center, retrospective study of 128 patients with advanced peritoneal metastasis who underwent CRS with intraoperative RBC transfusion between January 2018 and December 2024. Patients were stratified into low transfusion (≤4 units) and high transfusion (>4 units) groups. Propensity score matching (PSM) was applied to control for key covariates including CC score, operative time, blood loss, preoperative peritoneal cancer index (PCI), platelet count, age, and gender. Postoperative complications (Clavien–Dindo grade ≥ II) and overall survival (OS) were analyzed.

**Results:**

A total of 128 patients were included, among whom high intraoperative transfusion volume was significantly associated with higher CC score, greater blood loss, longer operative time, elevated preoperative PCI, and lower preoperative platelet counts. After propensity score matching, 48 patients were analyzed. The overall complication rate was 39.6%, with no significant difference between groups (*P* = 0.768). Hematologic recovery suggested transient improvement of postoperative anemia with higher transfusion volumes. However, long-term survival was significantly reduced in the high transfusion group, with 1-, 3-year OS rates of 42.7%, 18.3%, compared to 74.8%, 62.8%, and 50.2% in the low transfusion group (*P* = 0.019).

**Conclusions:**

Higher iPRBT may ease postoperative anemia but is associated with poorer long-term survival, without a clear link to postoperative complications. These findings support a more restrictive transfusion strategy in CRS/HIPEC.

## Introduction

Cytoreductive surgery (CRS) combined with hyperthermic intraperitoneal chemotherapy (HIPEC) has become a cornerstone in the treatment of peritoneal metastasis, particularly in patients with advanced malignancies. This approach is essential for improving survival in individuals with peritoneal carcinomatosis, offering the potential for long-term disease control (1, 2). However, CRS/HIPEC is a highly complex procedure that demands significant perioperative management, including the frequent need for blood transfusions. The intraoperative transfusion of red blood cells (RBCs) is commonly required to address blood loss during extensive surgical resection, but concerns regarding its impact on postoperative outcomes, particularly cancer recurrence, remain unresolved (3). Despite numerous studies linking blood transfusion to negative clinical outcomes, the precise effect of intraoperative packed red blood cell transfusion (iPRBT) on the prognosis of CRS/HIPEC patients has not been thoroughly explored. Existing studies have demonstrated a clear association between blood transfusions and adverse postoperative outcomes in various oncologic surgeries. For example, a multicenter study by et al. De la Garza Ramos (4) found that RBC transfusion was independently associated with an increased risk of complications, including sepsis and deep vein thrombosis, in patients undergoing spinal tumor surgery. Similarly, Swift et al. (5) reported that perioperative transfusion in gynecologic oncology patients increased the risk of composite morbidity and prolonged hospital stays. In the context of ampullary carcinoma, Yao et al. (6) also highlighted that intraoperative allogeneic RBC transfusion, particularly in higher volumes, independently predicted poorer survival outcomes following pancreatoduodenectomy.

The exact role of transfusion volume in influencing the long-term prognosis of CRS/HIPEC patients remains unclear, particularly in the context of this high-stress, high-risk surgical procedure. We employed propensity score matching (PSM), a statistical tool used to minimize confounding bias by matching patients with similar characteristics, to control for key covariates such as tumor burden, operative blood loss, and surgical invasiveness. While transfusion volume may serve as a marker of more advanced disease or increased surgical complexity, the use of PSM allowed for a more precise analysis of the impact of transfusion volume on both short-term and long-term outcomes. By carefully stratifying patients based on transfusion volumes, we sought to explore how higher transfusion volumes affect postoperative recovery and overall survival, contributing new evidence to this important area of oncologic surgery. Through this study, we aim to add clarity to the ongoing debate surrounding transfusion practices in CRS/HIPEC patients, emphasizing the need for a more nuanced, individualized approach to blood transfusion during these complex procedures. Understanding whether transfusion volume directly contributes to adverse outcomes or merely reflects underlying disease severity is crucial for optimizing clinical management and improving patient prognostication.

## Materials and methods

### Patients

This study was a single-center, retrospective analysis conducted in compliance with the Declaration of Helsinki and relevant clinical practice guidelines. The study protocol and informed consent were approved by the Ethics Committee of Jinan Central Hospital. The study period spanned from January 2018 to December 2024. Patients included in this study were those diagnosed with advanced malignancies and peritoneal metastasis who underwent CRS with intraoperative red blood cell transfusion.

Inclusion criteria for the study were: histopathological confirmation of peritoneal metastasis, availability of complete clinical data, and intraoperative red blood cell transfusion during CRS. Exclusion criteria included: concomitant end-stage diseases such as liver and renal failure, preoperative neoadjuvant chemotherapy or radiotherapy, patients with incomplete or missing clinical data, and those who received non-surgical or palliative surgery.

### Surgical procedures

Cytoreductive surgery combined with HIPEC has become a standard and effective approach for treating peritoneal metastatic tumors. Preoperative evaluation involved tumor marker assays (e.g., CEA, CA19-9, CA125) and contrast-enhanced CT scans of the chest and abdominal-pelvic region to assess distant metastases and tumor extent. During surgery, the choice of laparoscopic exploration or open surgery was based on the individual patient's condition. Laparoscopic exploration was primarily used to assess tumor resectability and formulate a personalized CRS plan. For open surgery, a midline laparotomy was performed for comprehensive exploration of the peritoneal cavity and peritoneal cancer index (PCI) evaluation.

The core surgical procedure, following the Sugarbaker technique, involved the systematic resection of affected organs and peritoneum, including omentectomy and resection of mesenteric mucinous tumors (7). Intraoperative red blood cell transfusion was administered using allogeneic suspended or washed packed red blood cells. All units were pre-storage leukoreduced according to institutional and national transfusion standards. Immediately following CRS, HIPEC was performed to maximize the cytotoxic effect on any residual disease.

### Outcomes and follow-up

The primary outcomes of this study were short-term and long-term prognoses. Short-term prognosis was defined by postoperative complications occurring within 30 days, classified according to the Clavien-Dindo classification (grade ≥ II) (8). Only cases meeting these criteria were included in the analysis. Long-term prognosis was assessed based on overall survival (OS), which is the duration from CRS treatment initiation to death. Postoperatively, patients received routine follow-up visits in outpatient clinics, and telephone follow-up was conducted every 3 months, with the last follow-up occurring on March 31, 2025.

### Measured outcomes

For the purpose of the study, patients were divided into two groups based on the volume of intraoperative red blood cell transfusion: ≤4 units as the “Low transfusion” group and >4 units as the “High transfusion” group. This threshold was selected because prior CRS/HIPEC studies have shown that transfusion volumes in the range of approximately 3–5 units are associated with worsening perioperative and oncologic outcomes (3, 9), and in our cohort >4 units corresponded to the upper one-third of transfusion exposure, representing patients with substantially higher transfusion requirements. To minimize potential confounding bias, PSM was performed using seven covariates: CC Score, operation time, blood loss, preoperative PCI, preoperative platelet count, gender, and age. The propensity score was estimated using a logistic regression model, followed by greedy matching (1:1 ratio without replacement) with a caliper width of 0.2 standard deviations of the logit of the estimated propensity score. The matched cohorts were compared in terms of demographic characteristics, hematological parameters, postoperative complications, and long-term oncological outcomes.

### Statistical analysis

Statistical analysis was performed using R software (version 4.3.3) and SPSS software (version 26.0; IBM Corporation, New York). Categorical variables were expressed as frequencies (*n*) and percentages (%) and analyzed using the chi-square test. For continuous variables with a normal distribution, means ± standard deviations (SD) were used, and group comparisons were conducted using independent sample *t*-tests. Non-normally distributed continuous data were expressed as median and interquartile range (P25, P75), and between-group comparisons were performed using the Mann–Whitney *U* test. Survival analysis was conducted using the Kaplan–Meier method and Cox regression.

## Results

### Comparison of patient clinical characteristics and surgical information

A total of 128 patients with advanced peritoneal metastasis from malignant tumors, who underwent CRS between January 2018 and December 2024, were included in the study. These patients were diagnosed with advanced peritoneal metastasis during surgery. The median age of the cohort was 61 years, with 35.94% of the patients being male. The median body mass index (BMI) was 22.4 kg/m^2^. The histological distribution of cancer types was as follows: pseudomyxoma peritonei (PMP) in 69 patients (53.91%), colorectal cancer in 21 patients (16.41%), gastric cancer in 16 patients (12.50%), ovarian cancer in 11 patients (8.59%), and other cancers in 10 patients (7.81%).

Among the 128 patients, 91 (71.09%) received ≤4 units of red blood cells (RBC), while 37 (28.91%) were transfused with >4 units., Between-group comparison analysis identified several factors that were significantly associated with increased RBC transfusion volume, including the CC Score (*P* = 0.032, *χ*^2^ = 4.616), operation time (*Z* = −3.261, *P* = 0.001), blood loss (*Z* = −4.439, *P* < 0.001), preoperative platelet count (Preop Platelets, *t* = −2.568, *P* = 0.011), and preoperative PCI (*t* = −4.028, *P* < 0.001) (Table 1). Covariate balance before and after matching was evaluated using standardized mean differences (SMDs), with detailed balance statistics provided in Supplementary Table S1.

**Table 1:** Incidence and risk factors for high-volume intraoperative RBC transfusion.

Variable		Overall (*n* = 128)	Low transfusion (*n* = 91)	High transfusion (*n* = 37)	*χ*^2^/*t*/*Z*	*P*-value
Gender	Male	46 (35.94%)	33 (25.78%)	13 (10.16%)	0.015	0.904
	Female	82 (64.06%)	58 (45.31%)	24 (18.75%)		
PAS	Yes	61 (47.66%)	45 (35.16%)	16 (12.50%)	0.406	0.524
	No	67 (52.34%)	46 (35.94%)	21 (16.41%)		
Diabetes	Yes	17 (13.28%)	13 (10.16%)	4 (3.13%)	0.276	0.599
	No	111 (86.72%)	78 (60.94%)	33 (25.78%)		
Hypertension	Yes	37 (28.91%)	27 (21.09%)	10 (7.81%)	0.089	0.765
	No	91 (71.09%)	64 (50.00%)	27 (21.09%)		
Intestinal obstruction	Yes	36 (28.13%)	22 (17.19%)	14 (10.94%)	2.429	0.119
	No	92 (71.88%)	69 (53.91%)	23 (18.75%)		
cardiovascular disease	Yes	11 (8.59%)	10 (7.81%)	1 (0.78%)	2.299	0.129
	No	117 (91.41%)	81 (63.28%)	36 (28.13%)		
Smoking history	Yes	16 (12.50%)	13 (10.16%)	3 (2.34%)	0.918	0.338
	No	112 (87.50%)	78 (60.94%)	34 (26.56%)		
Cancer type	PMP	69 (53.91%)	45 (35.16%)	24 (18.75%)	8.417	0.077
Colorectal cancer		21 (16.41%)	15 (11.72%)	6 (4.69%)		
Gastric cancer		16 (12.50%)	14 (10.16%)	2 (1.56%)		
Ovarian cancer		11 (8.59%)	11 (8.59%)	0 (0.00%)		
Other cancers		10 (7.81%)	6 (4.69%)	4 (3.13%)		
ASA score	2	87 (68.75%)	63 (49.22%)	24 (18.75%)	2.155	0.340
	3	38 (29.69%)	27 (21.09%)	11 (8.59%)		
	4	3 (2.34%)	1 (0.78%)	2 (1.56%)		
CC score	0 and 1	57 (44.53%)	46 (35.94%)	11 (8.59%)	4.616	0.032
	2 and 3	71 (55.47%)	45 (35.16%)	26 (20.31%)		
Operation time		278 (200,360)	253 (180,330)	360 (255,366.50)	−3.261	0.001
Blood loss		200 (100,600)	200 (50,400)	600 (200,1,000)	−4.439	0.000
Age		60.42 ± 11.45	60.06 ± 10.95	61.29 ± 12.74	−0.550	0.584
BMI		22.32 ± 3.81	22.54 ± 3.70	21.79 ± 4.06	1.000	0.319
Preop Hb		109.54 ± 18.08	109.39 ± 19.04	109.91 ± 15.72	−0.148	0.883
Preop RBC Count		3.77 ± 0.67	3.73 ± 0.69	3.87 ± 0.62	−1.051	0.295
Preop platelets		271.53 ± 108.86	255.82 ± 106.93	309.32 ± 105.4	−2.568	0.011
Preop Alb		35.08 ± 7.05	35.76 ± 7.45	33.39 ± 5.70	1.738	0.085
Preop PCI		20.81 ± 10.60	18.53 ± 10.38	26.40 ± 9.03	−4.028	0.000

PAS, previous abdominal surgery; ASA, American Society of Anesthesiologists; CC, completeness of cytoreduction; BMI, body mass index; Alb, albumin; PCI, peritoneal cancer index; Hb, hemoglobin. PMP refers to pseudomyxoma peritonei. Cancer types are classified as PMP and non-PMP malignancies. “Other Cancers” include cholangiocarcinoma, hepatocellular carcinoma, lung cancer, and others.

### PSM for comparative analysis

To minimize confounding factors, we performed PSM based on seven key variables: CC Score, operation time, blood loss, preoperative PCI, preoperative platelet count, age, and gender. This approach ensured comparability between the two groups. After matching, 24 patients from each group were selected, resulting in two well-matched cohorts. Post-matching analysis revealed no statistically significant differences in clinical and pathological characteristics between the two groups (Table 2).

**Table 2:** Baseline characteristics of patients after propensity score matching.

Variable		Overall (*n* = 48)	Low transfusion (*n* = 24)	High transfusion (*n* = 24)	*χ*^2^/*t*/*Z*	*P*-value
Gender	Male	16 (33.33%)	8 (16.67%)	8 (16.67%)	0.000	1.000
	Female	32 (66.67%)	16 (33.33%)	16 (33.33%)		
CC score	0 and 1	18 (37.5%)	11 (22.92%)	7 (14.58%)	1.422	0.233
	2 and 3	30 (62.5%)	13 (27.08%)	17 (35.42%)		
Operation time		281.10 ± 98.51	274.79 ± 91.12	287.42 ± 106.98	−0.440	0.662
Blood loss		269 (206.75,324.25)	269 (194,324.25)	268.5 (213.5,339.75)	−0.675	0.499
Age		59.98 ± 12.31	58.63 ± 10.52	61.33 ± 13.96	−0.759	0.452
BMI		22.43 ± 4.28	23.18 ± 4.39	21.68 ± 4.12	1.216	0.230
Preop Hb		112.96 ± 15.43	114.54 ± 13.47	111.38 ± 17.32	0.707	0.483
Preop platelets		277.91 ± 101.12	268.27 ± 103.62	286.75 ± 100.15	−0.615	0.542
Preop lymphocyte count		1.22 ± 0.43	1.31 ± 0.39	1.13 ± 0.46	0.077	0.149
Preop RBC count		3.95 ± 0.53	3.96 ± 0.47	3.95 ± 0.59	0.352	0.988
Preop PCI		24.25 ± 8.72	24.46 ± 8.86	24.04 ± 8.76	0.164	0.871

CC, completeness of cytoreduction; BMI, body mass index; Hb, hemoglobin; PCI, peritoneal cancer index.

### Short-term comparison of hematological parameters between low and high transfusion groups

**Figure 1:**
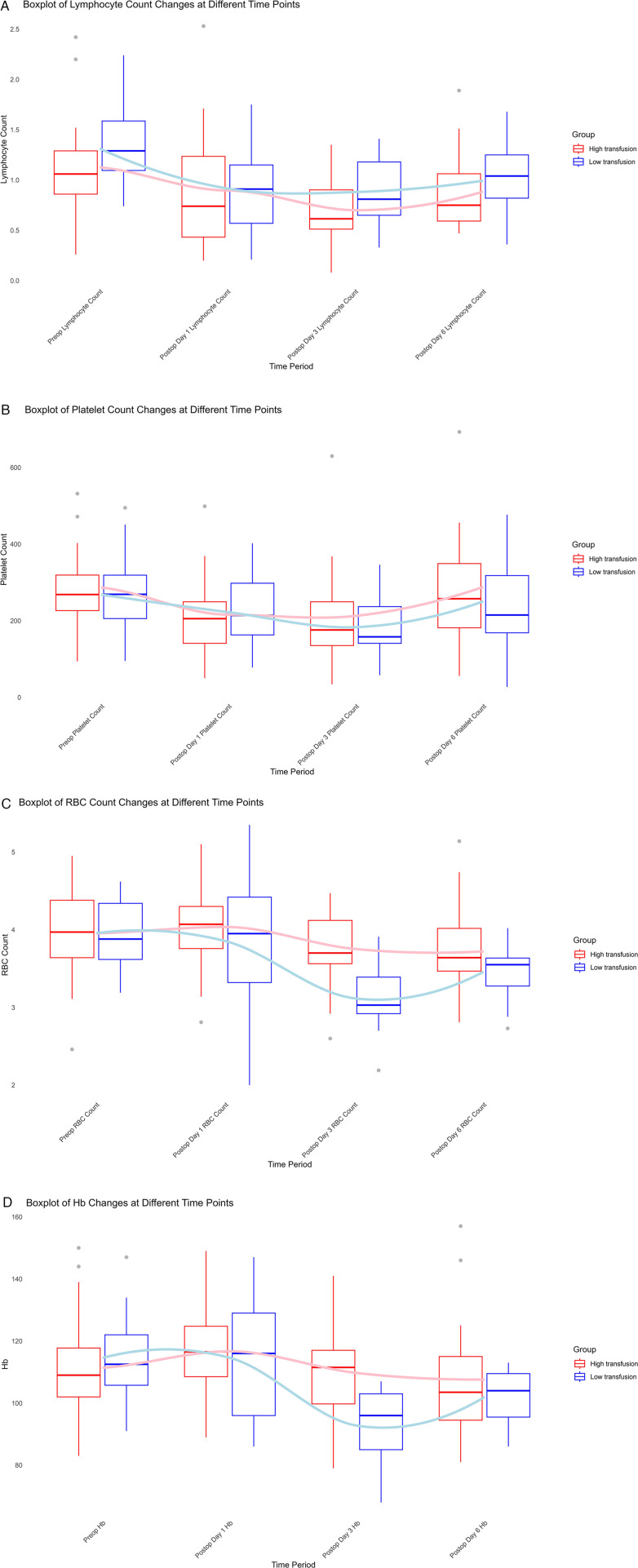
Perioperative hematologic changes in low- and high-transfusion groups. **(A)** Lymphocyte count, **(B)** platelet count, **(C)** red blood cell (RBC) count, and **(D)** hemoglobin (Hb) levels from preoperative baseline to postoperative day 6. Both lymphocyte and platelet counts decreased initially and then recovered without significant intergroup differences. In contrast, RBC and Hb showed a more pronounced decline in the low transfusion group, with significant differences observed on postoperative day 3 (*P* < 0.05).

Analysis of the two groups showed that both lymphocyte count and platelet count decreased initially and then increased from preoperative levels to postoperative day 6, with no significant differences between the groups (Figures 1A,B). Regarding red blood cell (RBC) count, the low transfusion group demonstrated an initial decline followed by recovery from postoperative day 1 to day 6. In contrast, the high transfusion group showed a continuous decline, albeit at a slower rate. A statistically significant difference was observed between the groups on postoperative day 3 (*P* = 0.001, *t* = −3.664), with values of 3.12 ± 0.43 in the low transfusion group and 3.75 ± 0.53 in the high transfusion group (Figure 1C). Hemoglobin levels followed a similar trend to RBC count, with a less pronounced decline in the high transfusion group. A significant difference was noted on postoperative day 3 (*P* = 0.002, *t* = −3.415), with values of 92.73 ± 12.17 in the low transfusion group and 109.69 ± 15.18 in the high transfusion group (Figure 1D). These findings suggest that higher intraoperative RBC transfusion may have a potential beneficial effect on the improvement of postoperative anemia.

### Postoperative complications in short-term outcomes

Postoperative complications occurring within 30 days after CRS were evaluated and categorized according to the Clavien–Dindo classification (grade ≥ II). Among the 48 propensity-matched patients, 19 (39.58%) experienced at least one postoperative complication. The most frequent complications included abdominal infection (18.75%), respiratory complications (16.67%), and liver dysfunction (10.42%). Less common events involved circulatory disorders, renal failure, small bowel obstruction, and diarrhea, each occurring in 6.25% of cases. The overall incidence of complications showed no significant difference between the low and high transfusion groups (*P* = 0.768). Likewise, no statistically significant differences in liver dysfunction (*P* = 0.637), respiratory complications (*P* = 0.121), or abdominal infections (*P* = 0.712) between the two groups (Table 3).

**Table 3:** Postoperative complications following CRS treatment.

Variable	Overall (*n* = 48)	Low transfusion (*n* = 24)	High transfusion (*n* = 24)	*P*-value
All complications				0.768
Yes	19 (39.58%)	9	10	
No	29 (60.42%)	15	14	
Liver dysfunction				0.637
Yes	5 (10.42%)	3	2	
No	43 (89.58%)	21	22	
Respiratory system				0.121
Yes	8 (16.67%)	2	6	
No	40 (83.33%)	22	18	
Abdominal infection				0.712
Yes	9 (18.75%)	4	5	
No	39 (81.25%)	20	19	
VTE	2 (4.17%)	2	0	
Circulatory system	3 (6.25%)	3	0	
Renal failure	3 (6.25%)	1	2	
Small bowel obstruction	3 (6.25%)	1	2	
Gastroparesis	2 (4.17%)	1	1	
Anastomotic leak	2 (4.17%)	0	2	
Bladder leak	1 (2.08%)	0	1	
SSI	2 (4.17%)	0	2	
Diarrhea	3 (6.25%)	2	1	
Death	3 (6.25%)	1	2	

VTE, venous thromboembolism; SSI, surgical site infection.

### Long-term outcomes

**Figure 2:**
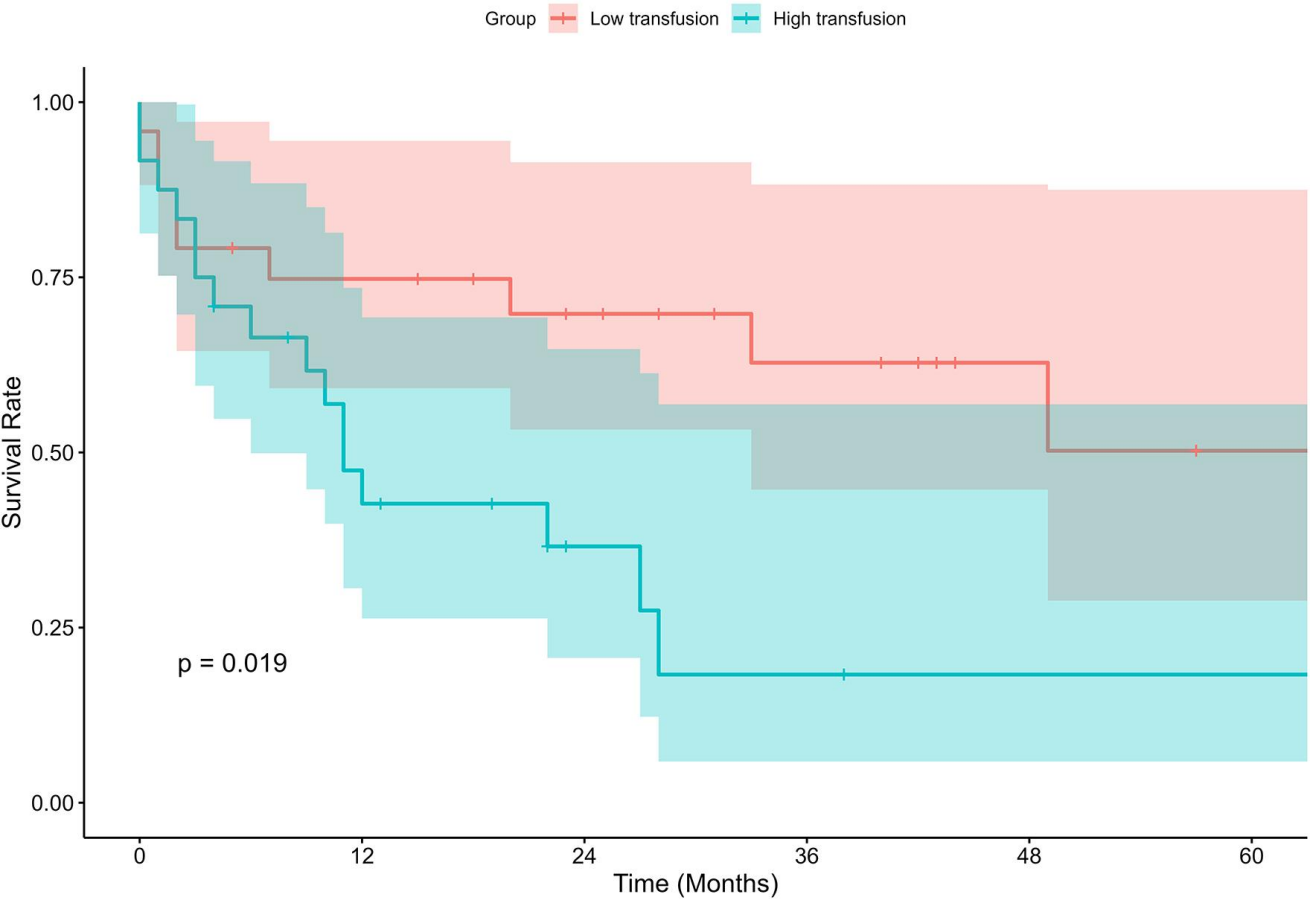
Kaplan–Meier survival curves of low- and high-transfusion groups.

All 48 patients were followed up for survival, with a median follow-up duration of 16.5 months (25%, 75% percentiles: 4, 32 months). In the low transfusion group, the estimated 1-, 3-, and 5-year OS rates were 74.8%, 62.8%, and 50.2%, respectively. In contrast, the high transfusion group exhibited significantly lower survival probabilities at each time point, with corresponding 1-, 3-year OS rates of 42.7%, 18.3%. Kaplan–Meier survival analysis revealed a statistically significant difference in OS between the two groups (*P* = 0.019), as illustrated in the survival curves (Figure 2).

Additionally, survival analysis using Cox regression in the 128 patients identified three factors significantly associated with survival: intraoperative RBC transfusion (OR 1.082, 95% CI 1.001–1.161, *P* = 0.047), CC Score (OR 0.382, 95% CI 0.180–0.885, *P* = 0.023), and Albumin (OR 0.900, 95% CI 0.836–0.968, *P* = 0.004) (Table 4).

**Table 4:** Survival analysis using Cox regression in 128 patients.

Variable	*Β*	OR (95% CI)	*P*-value
Intraoperative RBC transfusion	0.639	1.082 (1.001–1.161)	0.047
Age	−0.006	0.994 (0.957–1.032)	0.752
Gender	0.318	1.374 (0.614–3.072)	0.439
BMI	0.041	1.042 (0.954–1.138)	0.359
CC score	−1.193	0.382 (0.180–0.885)	0.023
Albumin	−0.106	0.900 (0.836–0.968)	0.004
ASA score	0.002	1.002 (0.997–1.006)	0.471
Preop Hb	0.009	1.009 (0.982–1.035)	0.525

## Discussion

Despite the growing body of literature suggesting an association between blood transfusion and adverse outcomes in oncologic surgery, limited data exist specifically within the setting of CRS/HIPEC—a context characterized by substantial physiological stress, extensive surgical trauma, and high transfusion demands (3, 10, 11). In this study, iPRBT was found to be significantly associated with higher CC scores, elevated PCI, prolonged operative duration, greater intraoperative blood loss, and lower preoperative platelet counts—factors reflective of more extensive disease burden and surgical complexity. Recognizing the challenge of disentangling the prognostic impact of transfusion from that of underlying tumor biology, we introduced an intra-patient transfusion stratification metric and applied propensity score matching based on key surgical and oncologic parameters. This approach allowed for a more refined evaluation of transfusion effects independent of disease invasiveness. Hematologic trajectories suggested that higher transfusion volumes may transiently improve postoperative anemia without increasing short-term morbidity.

Despite this short-term neutrality, patients receiving lower transfusion volumes demonstrated superior overall survival at 1-, 3-, and 5-year intervals, suggesting that transfusion may exert long-term deleterious effects beyond its role as a marker of surgical difficulty. These findings align with concerns raised in broader oncologic surgery literature, yet data specific to CRS/HIPEC—an environment uniquely characterized by profound physiological stress and high transfusion demands—remain scarce (9, 12, 13). While the observational nature of this study limits the ability to establish causality, our results provide novel evidence suggesting that iPRBT may influence oncologic outcomes. This emphasizes the need for cautious, indication-based transfusion practices in this high-risk surgical population. Further research is warranted to determine whether transfusion is simply a reflection of disease severity or a direct modulator of prognosis.

Building on previous studies linking transfusion to adverse outcomes in oncologic surgery, the role of blood transfusion in influencing patient prognosis remains a critical area of research, particularly within the unique physiological and surgical context of CRS/HIPEC procedures. Fisher et al. demonstrated that iPRBT, particularly when normalized by the PCI, is an independent predictor of worse OS and recurrence-free survival (RFS). Higher transfusion volumes were associated with poorer outcomes, a trend most pronounced in patients with high-grade mucinous appendiceal neoplasms, but also observed across other tumor types (3). Similarly, Boateng Kubi et al. reported that even low-volume perioperative allogeneic blood transfusion (PABT) was independently associated with increased risks of both infectious and non-infectious postoperative complications, including pleural effusion, hemorrhage, and reoperation. Moreover, transfusing more than five RBC units significantly worsened postoperative outcomes. PABT was identified as an independent predictor of poorer survival in patients with appendiceal or colorectal peritoneal carcinomatosis (9). Additionally, a study of 67 patients undergoing CRS-HIPEC for colorectal peritoneal metastasis found that blood transfusion independently increased morbidity (14). A separate study of 936 peritoneal cancer patients further demonstrated that perioperative blood transfusion, particularly massive blood transfusion (≥5 units), significantly worsened postoperative outcomes, including increased complications and longer hospital stays, particularly in patients with colorectal cancer peritoneal carcinomatosis. Massive blood transfusion was also recognized as an independent predictor of poorer long-term survival, as evidenced by significantly reduced overall survival in patients with colorectal cancer and pseudomyxoma peritonei (15).

The mechanisms by which blood transfusion negatively affects patient outcomes, particularly in relation to cancer progression and recurrence, have been widely investigated. Transfusion-related immunomodulation (TRIM) is a key concept suggesting that allogeneic blood transfusions can alter the recipient's immune system in a way that may promote tumor spread and recurrence. Specifically, transfusion has been shown to induce a Th2-dominant cytokine profile, with elevated levels of IL-4, IL-10, and TGF-β, which suppress cytotoxic immune responses. This shift in immune response impairs the body's ability to recognize and eliminate tumor cells (10, 16, 17). Moreover, transfusion has been associated with decreased activity of natural killer (NK) cells and lymphokine-activated killer cells, further compromising anti-tumor immunity (18). Macrophages play a central role in mediating the immune modulation caused by red blood cell transfusion, primarily through phagocytosis of transfused cells (19). The increased risk of infection and cancer recurrence post-transfusion can also be attributed to the impaired function of other antigen-presenting cells (APCs), including NK cells, which are essential for maintaining cell-mediated immunity (20). Additionally, storage-induced lesions in red blood cells release bioactive substances, such as free hemoglobin, prostaglandins, isoprostanes, and microRNAs (e.g., miR-451a), which exacerbate immune suppression and endothelial dysfunction (21). While leukoreduction has been utilized to reduce leukocyte-dependent immunologic reactions, its impact on cancer-related outcomes remains uncertain. On the other hand, gamma irradiation of blood products has been shown to reduce immunosuppressive effects by decreasing regulatory T cell populations and lowering IL-10 levels (22, 23). Collectively, these mechanisms emphasize the complex immunosuppressive effects of blood transfusion, particularly with allogeneic and stored red blood cells, which may promote tumor progression and recurrence by altering the tumor microenvironment. All intraoperative RBC units in our cohort were pre-storage leukoreduced, yet residual immunomodulatory effects may still influence long-term outcomes.

The limitations of this study cannot be overlooked. First, while PSM was employed to minimize confounding factors, residual biases may still persist due to the nature of the observational design. Unmeasured variables, such as the timing and method of red blood cell transfusion or the specific transfusion protocols used, could influence the results. Additionally, the retrospective nature of this study limits the ability to establish causal relationships between transfusion volume and patient outcomes. The data relies on historical patient records, which may have inherent limitations in accuracy and completeness. Furthermore, the potential impact of other blood products administered during surgery, as well as the role of allogeneic blood transfusions (ABT) throughout the entire perioperative period, warrant further consideration. In the present study, only intraoperative packed red blood cell transfusions were analyzed, and postoperative transfusions were not captured in the exposure definition, which may underestimate the total transfusion burden. Further prospective, multi-center studies with longer follow-up are required to validate these findings and better understand the mechanisms underlying the relationship between transfusion volume and postoperative outcomes in CRS.

## Conclusion

While higher iPRBT volumes may temporarily mitigate anemia, they are associated with significantly poorer long-term survival in CRS/HIPEC patients. These findings underscore the importance of judicious transfusion practices and suggest that minimizing intraoperative transfusion volume may improve prognosis. Prospective, multicenter studies are warranted to validate these results.

## Data Availability

The original contributions presented in the study are included in the article/Supplementary Material, further inquiries can be directed to the corresponding author.

## Supplementary material

Supplementary Table S1 Standardized mean differences (SMD) before and after propensity score matching.
