# Supplementary material for: Impact of intraoperative packed red blood cell transfusion volume on prognosis in CRS: a propensity-matched study
Source: Front Surg. 2026 Jan 12;12:1688163. doi: 10.3389/fsurg.2025.1688163 (PMC12832702; doi:10.3389/fsurg.2025.1688163)
Supplement: Supplementary Table S1 — Standardized mean differences (SMD) before and after propensity score matching. [file Table1.docx]

Supplementary Table S1. Standardized Mean Differences (SMD) Before and After Propensity Score Matching

| Variable | SMD_before | SMD_after |
| --- | --- | --- |
| Gender | 0.013 | 0.000 |
| CC Score | 0.146 | 0.044 |
| Operation Time | 0.619 | 0.130 |
| Blood Loss | 0.982 | 0.116 |
| Age | 0.105 | 0.024 |
| BMI | 0.193 | 0.159 |
| Preop Hb | 0.030 | 0.209 |
| Preop Platelets | 0.509 | 0.185 |
| Preop Lymphocyte Count | 0.223 | 0.102 |
| Preop RBC Count | 0.215 | 0.005 |
| Preop PCI | 0.815 | 0.048 |
